# Nonisothermal Crystallization of Surface-Treated Alumina and Aluminum Nitride-Filled Polylactic Acid Hybrid Composites

**DOI:** 10.3390/polym11061077

**Published:** 2019-06-21

**Authors:** Zelalem Lule, Jooheon Kim

**Affiliations:** School of Chemical Engineering & Materials Science, Chung-Ang University, Seoul 156-756, Korea; zochernet@gmail.com

**Keywords:** hybrid composites, crystallinity, solution casting, differential scanning calorimetry

## Abstract

This work investigates the nonisothermal crystallization and melting behavior of polylactic acid (PLA), filled with treated and untreated alumina and nano-aluminum nitride hybrid composites. Analysis by attenuated total reflectance Fourier transform infrared spectroscopy revealed that the treated fillers and the PLA matrix developed a good interaction. The crystallization and melting behaviors of the PLA hybrid composites were investigated using differential scanning calorimetry showed that the degree of crystallinity increased with the addition of hybrid fillers. Unlike the untreated PLA composites, the complete crystallization of the treated PLA hybrid composites hindered cold crystallization during the second heating cycle. The crystallization kinetics studied using the Avrami model indicated that the crystallization rate of PLA was affected by the inclusion of filler particles. X-ray diffraction analysis confirmed crystal formation with the incorporation of filler particles. The inclusion of nano-aluminum nitride (AlN) and the increase in the crystallinity led to an improvement of the storage modulus.

## 1. Introduction

Recent increasing production of biodegradable plastic materials has raised environmental concerns about the large-scale production of nonbiodegradable and nonrecyclable petroleum-based polymers. Polylactic acid (PLA) is a widely utilized biodegradable polymer because of its good mechanical properties, high stiffness, high transparency, excellent printability, and good processability [1,2]. Currently, PLA is mainly produced through the polymerization of lactides from renewable sources such as potato, corn, and bagasse. PLA can be used industrially and academically in biomedical, food packaging, and electronics research fields [3]. However, the application of PLA-based materials is limited to the aforementioned specific sectors because of its unfavorable properties, which include slow crystallization, high gas diffusion, and brittleness [4]. The crystallization of PLA has been improved through the fabrication of composites incorporating carbon-based materials [2,5], clays [6,7], and ceramic fillers [8,9,10].

PLA is a semicrystalline polymer, the morphological, mechanical, and physical properties of which are controlled by its crystallization behavior [11]. Because of the chiral nature of lactic acids, the lactides, the building block of PLA, exist in *l*-lactide and *d*-lactide forms. Depending on the amount of *l*-lactide and *d*,*l*-lactide components, PLA can crystallize in three crystal forms: α, β, and γ forms [4,12]. Among these crystal forms, the α-phase, which occurs during melt or cold crystallization, is the most stable. The brittle nature of PLA is a consequence of its low glass-transition temperature, which is another factor that limits its application [13]. Hence, many researchers have suggested that increasing the extent and rate of crystallization would change the microstructure of the matrix and expand its practical application prospects. Park et al. [2] fabricated a PLA/carbon nanotube (CNT) composite with enhanced mechanical properties stemming from a substantial improvement in the crystallization kinetics as a result of the addition of CNTs. Li et al. [14] confirmed that improvements in the dynamic mechanical properties of a PLA composite filled with microcrystalline cellulose (MCC) at a very low MCC content are attributable to an increase in the crystallinity and crystallization rate.

In our previous work [15], we suggested that alumina affects the crystallization behavior of polybutylene succinate (PBS). However, the effect of alumina on the crystallization of the PBS matrix has not been discussed in depth. Numerous studies have confirmed the effect of alumina on the melting and crystallization properties of polymer matrices. For example, Kuo et al. [16] concluded that the inclusion of nanosized alumina restricted the chain mobility of poly(ether ether ketone) (PEEK), which played a dominant role in increasing its crystallization time. In addition, Mosavian et al. [17] used the Avrami model to study the nonisothermal crystallization kinetics of high-density polyethylene (HDPE)/alumina composites at different cooling rates. Their results revealed that the crystallization peak broadened and shifted to lower temperatures with increasing cooling rate.

In the present study, we synthesized a PLA composite reinforced with alumina and nanosized aluminum nitride (AlN) hybrid fillers via a solution casting process. In our previous study, the inclusion of nanosized AlN improved the storage modulus of the PBS nanocomposite [18]. Consequently, we expected the combined effect of alumina and AlN to improve both the crystallization behavior and the stiffness of the resultant PLA hybrid composite. The surface properties of the hydrophobic PLA and the relatively hydrophilic alumina/AlN hybrid fillers are dissimilar. Consequently, the fillers were surface treated before being mixed with the PLA to improve their interaction with the matrix [19]. The composite was then characterized using differential scanning calorimetry (DSC), X-ray diffraction (XRD), and dynamic mechanical analysis (DMA). 

## 2. Experimental

### 2.1. Filler Surface Treatment

Prior to the fabrication of the PLA hybrid composite, the filler materials were surface treated. The alumina particles were surface functionalized with poly(maleic acid) using a modified version of a procedure reported elsewhere [20]. First, 0.04 M maleic acid (MA) solution was prepared; alumina (Sigma-Aldrich, Seoul, South Korea) particles were subsequently added to this solution, and the resultant mixture was stirred at room temperature for 4 days. The alumina particles were then separated from the solution and air dried for 1 day. The MA molecules that adsorbed onto the alumina particles (Al–MA) were allowed to polymerize at 80 °C in the presence of 1-octadecene monomer as a solvent in a three-necked flask equipped with a nitrogen gas inlet, condenser, and thermometer. After the polymerization temperature was reached, azobisisobutyronitrile (AIBN) initiator was added to the mixture and the system was maintained at 80 °C for 3 h under a nitrogen atmosphere. The surface-functionalized alumina particles (Al–poly(MA)) were separated from the mixture and air dried for 24 h. In addition, AlN (Sigma-Aldrich, Seoul, South Korea) nanoparticles were also stirred and sonicated with dimethylformamide (DMF). The AlN solution was centrifuged for 30 min at 10,000 rpm to separate the lighter particles with a relatively similar size. 

### 2.2. PLA Hybrid Composite Fabrication

The PLA (PLLA homopolymer, Jae Youn Chemical Co., Ltd., Gangwon-do, South Korea) pellets were oven dried at 50 °C for 24 h to eliminate surface moisture, which can otherwise lead to void formation. The PLA pellets were placed in chloroform solvent and stirred for 4 h at room temperature. In a separate flask, the proper amounts of alumina (18 wt %, 28 wt %, 38 wt %, or 48 wt %) and AlN (2 wt %) were mixed in chloroform and sonicated for 30 min. Subsequently, the hybrid filler mixture was transferred into the PLA solution and the resultant mixture was allowed to mix for 90 min. The solution was then poured into a Petri dish when the solution became viscous, indicating the formation of the PLA hybrid composite. The Petri dish was maintained at room temperature while covered with a lid (with a small opening) so as to decrease the solvent evaporation rate. The dried PLA hybrid composite films (Table 1) were subsequently collected for further analysis. Neat PLA was also synthesized in the same manner and used as a control. 

## 3. Characterization

The raw and treated alumina particles were characterized using Fourier transmission infrared spectroscopy (FT-IR, Nicolet iS5, Thermo Fisher Scientific, Seoul, Korea) to confirm whether the poly (maleic acid-1-octadecene) was effectively grafted on the alumina surface. Attenuated total reflectance Fourier transform infrared spectroscopy (ATR-FTIR, Nicolet 6700, Thermo Scientific, Seoul, Korea) analysis was conducted to study the surface of the PLA composites. The analysis was conducted in a wide frequency range from 4000 to 400 cm^−1^ at a resolution of 4.0 cm^−1^.

The melting and crystallization behavior of the PLA hybrid composites were investigated by DSC (KEP Tech., Mougins, France). The investigation was carried out in a heating–cooling–heating cycle under a nitrogen atmosphere. The specimens were heated from 30 to 220 °C at a rate of 10 °C/min and then maintained at 220 °C for 1 min to remove the thermal history. The samples were subsequently cooled to 30 °C at various cooling rates (5, 10, and 20 °C/min). Finally, the samples were reheated to 220 °C at the heating rate corresponding to the previous cooling rate. The percentage crystallinity (*X_c_*) of the PLA was calculated from the second heating cycle using the following Equation:

(1) $$X_{C}\left(\%\right)=\frac{∆H_{m}-∆H_{cc}}{∆H_{m}^{O}\times w_{PLA}}\times 100$$

where (*X_c_*) is the degree of crystallinity of PLA; $∆H_{m}^{O}$ is the enthalpy of fusion of 100% crystalline PLA (93 J/g) [21]; ∆*H_cc_* and ∆*H_m_* are the enthalpies of cold crystallization and melting, respectively; and *w_PLA_* is the weight fraction of PLA in the hybrid composites.

The XRD patterns of the alumina particles and PLA composites were collected using an X-ray diffractometer (XRD, New D8 Advance, Bruker AXS) equipped with a Cu-Kα radiation source. The XRD patterns were collected over the scanning-angle range 5° ≤ 2θ ≤ 80°. The storage modulus of the PLA hybrid composite films was measured using a dynamic mechanical analyzer (Triton Tech., UK). The analysis was performed in the temperature range from −50 to 165 °C at a frequency of 1 or 10 Hz.

## 4. Results and Discussion

### 4.1. Surface Characterization

**Figure 1:**
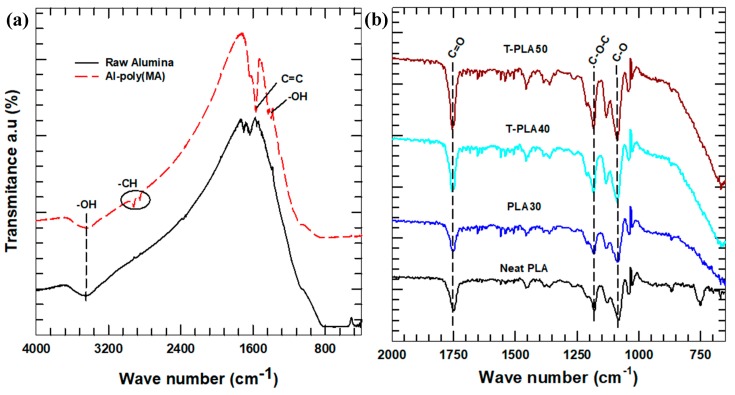
(**a**) FTIR spectra of the raw and treated alumina; (**b**) Attenuated total reflectance Fourier transform infrared spectroscopy (ATR-FTIR) of the polylactic acid (PLA) composites.

The surface of the alumina particles, the neat PLA, and the PLA composites were analyzed by FTIR. The corresponding FTIR spectral curves are shown in Figure 1. Some peaks disappeared and new ones appeared after the particles were surface treated. The sharp peaks at 2825 and 2927 cm^−1^ in the FTIR spectrum of the treated alumina are assigned to the stretching vibrations of –CH_3_ and –CH_2_ functional groups, respectively [22]. In addition, a C–O peak that appeared because of the interaction of surface oxygen and atmospheric carbon disappeared after the particles were treated. In general, the surface of the alumina was well functionalized. ATR-FTIR analysis is sensitive toward organic functional groups and is helpful in eliminating the effects of surface moisture, which leads to the appearance of unnecessary peaks in the spectra [11]. In the case of the PLA spectrum (Figure 1b), main peaks at 1750, 1180, and 1084 cm^−1^ are attributed to C=O, C–O–C, and C–O stretching vibrations, respectively [23]. The incorporation of alumina leads to the disappearance of two bending modes at 669 and 750 cm^−1^, revealing the effect of fillers on the chemical structure of the matrix, which is attributed to the amorphous phase of PLA [24]. This result could mean that the PLA hybrid composites have higher crystallinity when compared to the neat PLA.

### 4.2. Nonisothermal Melt and Crystallization Behavior

#### 4.2.1. Crystallization Behavior

**Figure 2:**
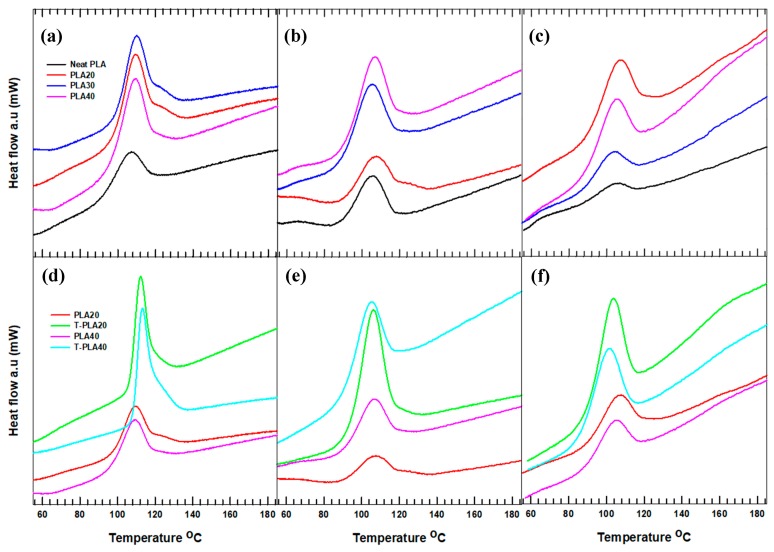
Crystallization thermographs of neat and hybrid composites: the effects of filler loading at cooling rates of (**a**) 5 °C/min, (**b**) 10 °C/min, and (**c**) 20 °C/min and the effects of filler treatment at cooling rates of (**d**) 5 °C/min, (**e**) 10 °C/min, and (**f**) 20 °C/min.

The melting and crystallization behavior of the neat and hybrid composites were analyzed using DSC at three different heating and cooling rates. The crystallization behavior of the hybrid composite samples was studied using the first cooling cycle recorded at various cooling rates after the thermal history had been removed via the first heating. The corresponding crystallization curves at cooling rates of 5, 10, and 20 °C/min are shown in Figure 2. From Figure 2a–c, the crystallization curve is broad and has a higher peak height at a lower cooling rate, indicating a decrease in the enthalpy of crystallization (Δ*H_c_*) with increasing cooling rate. This result confirms that, at lower cooling rates, the samples have sufficient time to form crystals, whereas increasing the cooling rate forces the crystallization process to occur faster, without complete crystal formation. However, the crystallization peak (*T*_c_) of the composites synthesized with treated filler exhibited a sharper peak and a higher enthalpy of crystallization compared with their equivalent composites with untreated filler loadings (Figure 2d–f). This observation confirms that the incorporation of treated filler led to the easier formation of crystals in T-PLA20 and T-PLA40 at the early stage of crystallization. 

To further explain the aforementioned crystallization mechanism, the relative crystallinity (*X_t_*) as a function of temperature (*T*) was determined using the integral method, as follows:

(2) $$X_{t}=\frac{\int_{T_{o}}^{T}\left(\frac{dH_{C}}{dT}\right)dT}{\int_{T_{o}}^{T_{\infty }}\left(\frac{dH_{C}}{dT}\right)dT}$$

**Figure 3:**
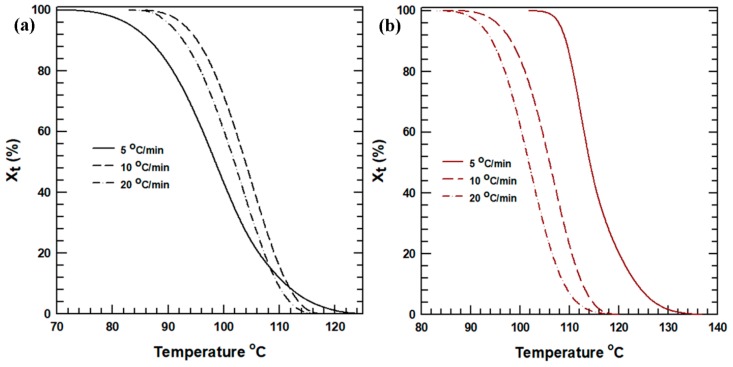
Relative crystallinity curve as a function of temperature: (**a**) neat PLA and (**b**) T-PLA50.

The relative crystallinity curve of the neat PLA and T-PLA50 plotted as a function of the crystallization temperature is shown in Figure 3 and Figure S1. All of the curves exhibit a sigmoidal shape with higher early crystallization and slower final crystallization. At a cooling rate of 5 °C/min, the crystallization started (onset) at a higher temperature and completed (offset) at a lower temperature compared with the crystallization processes at the other cooling rates. These results clarify the appearance of a broader crystallization curve (Figure 2a–c) at lower cooling rates. However, the crystal formation temperature range in T-PLA50 was similar irrespective of the cooling rate, confirming that the interaction of the treated hybrid fillers and the PLA matrix influenced the crystallization rate and mechanism. In general, we concluded that the inclusion of treated hybrid fillers affected the crystallization process of the PLA composites at different cooling rates.

#### 4.2.2. Melting and Cold-Crystallization Properties

**Figure 4:**
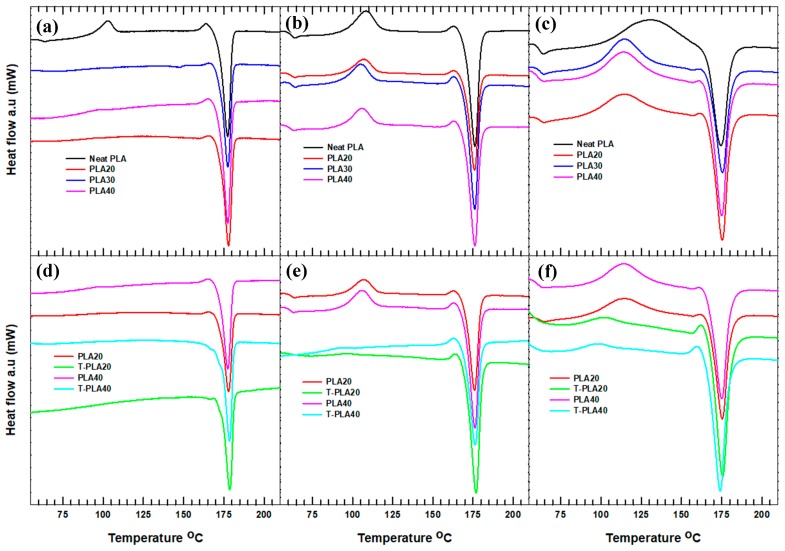
Cold crystallization and melting thermographs of neat and hybrid composites: effect of filler loading at heating rates of (**a**) 5 °C/min, (**b**) 10 °C/min, and (**c**) 20 °C/min; effect of filler treatment at heating rates of (**d**) 5 °C/min, (**e**) 10 °C/min, and (**f**) 20 °C/min.

The melting and cold-crystallization points were recorded from the second DSC heating cycle. The first heating cycle was performed at the same 10 °C/min heating rate for all of the samples to control the effect of the heating cycle on the subsequent steps. The cooling and second heating were then carried out at the selected rates. The second heating cycle thermographs for the neat PLA and its hybrid composites are shown in Figure 4. The melting temperature did not substantially change for any of the specimen samples with the inclusion of different filler loading of hybrid fillers (Figure 4a–c), filler treatments (Figure 4d–f), or heating rates. 

For PLA composites filled with untreated hybrid fillers, the increase in the heating rate lead to the appearance of a cold-crystallization curve accompanied with an increase in the Δ*H_cc_*. The disappearance of cold crystallization peaks (*T_cc_*) at lower heating rates was due to the slow and complete crystallization process prior to the heating cycle. At lower cooling rates, the samples have sufficient time to crystallize (Figure 2a–c) and inhibit the formation of a cold crystallization peak. On the contrary, at a heating rate of 20 °C/min, the cold crystallization peak becomes broader with increasing Δ*H_cc_*. In particular, the *T_cc_* of the neat PLA heated at 20 °C/min shifts to higher temperatures and becomes broad, with the offset cold-crystallization temperature continuing until the onset melting temperature. This scenario occurred mainly due to the previous incomplete crystallization (Figure 2c) related to the fast cooling rate. The increase in the enthalpy of cold crystallization with increasing heating rate leads to a decrease in the degree of crystallinity (*X_c_*) for each sample with the same filler content. In addition, the hybrid composites filled with untreated fillers show a decrease in the degree of crystallinity when the heating rate is increased from 5 to 20 °C/min (Table 2).

The effect of filler treatment on the cold crystallization and melting behavior of the PLA composites was similarly investigated (Figure 4d–f). Because of the slower crystallization rate of the composite with treated filler particles, the cold-crystallization temperature decreased compared with that of the PLA composites with untreated fillers. The increase in the heating/cooling rate does not induce a clear change in the cold-crystallization temperature of the treated samples. At 20 °C/min, the cold-crystallization temperature for both T-PLA20 and T-PLA40 was lower than that of the corresponding untreated PLA composite samples. Moreover, the Δ*H_cc_* decreased and the Δ*H_m_* increased, which is associated with the restriction of polymer chain mobility due to the interaction of the treated alumina and the PLA matrix [16]. As a result, the degree of crystallinity for the given composites filled with treated filler materials did not substantially change with increasing heating rate.

In general, samples that crystallize at a lower cooling rate have sufficient time to undergo complete crystallization. Hence, either no cold-crystallization peak or only a small cold-crystallization peak would be observed when the sample is heated. By contrast, if the sample is cooled at a high cooling rate, it would either uncrystallize or undergo partial crystallization, leading to the appearance of a cold-crystallization peak when the sample is heated.

### 4.3. Nonisothermal Crystallization Kinetics

**Figure 5:**
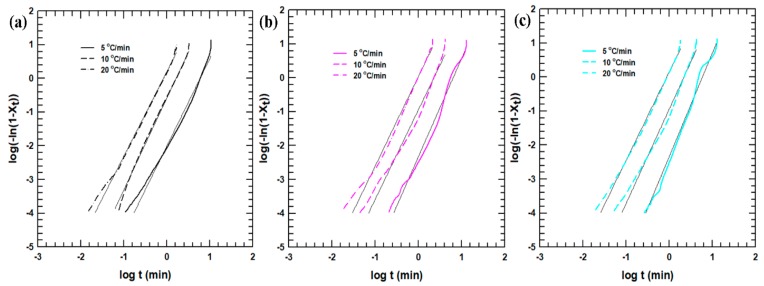
Avrami curves for (**a**) neat PLA; (**b**) PLA40; and (**c**) T-PLA40.

The nonisothermal crystallization kinetics of the PLA hybrid composites were estimated using the Avrami model (Equation (3a)) [25,26]. Although this model has usually been applied to isothermal crystallization processes, it can also be used to characterize nonisothermal kinetics. To analyze the crystallization kinetics, the crystallization temperature was converted to time (Equation (3b)). The relative crystallinity was also plotted as a function of time to investigate the crystal formation at different stages (Figure S2). The results show that the crystallization process occurred much faster with an increase in the cooling rate. This result further reiterates that the slow crystallization processes that occurred at lower cooling rates played a substantial role in the completion of crystal formation:

(3a) $$\log\left[-\ln\left(1-X_{t}\right)\right]=n\;log\;\left(t\right)+\log k$$

(3b) $$t=\frac{\left|T_{o}-T\right|}{\psi }$$

where *n* is the Avrami exponent; *k* is the rate constant; and *ψ* is the cooling rate. The slope and the intercept of the linearized curve of $\log\left[-\ln\left(1-X_{t}\right)\right]$ vs. log(t) provide the values of *n* and *k*, respectively. The Avrami plots at different cooling rates are shown in Figure 5 and Figure S3, and the slope and intercept of the linearized curve are tabulated in Table 3. The value of *n* varies with the crystallization mechanism and growth geometry, whereas the value of *k* is associated with the crystallization rate [27]. Irrespective of the filler loading or filler treatment, the PLA hybrid composites that did not exhibit a sufficiently large value of *n* did not show a uniform change. However, the composites with higher filler loadings had relatively higher *n* values compared with the neat PLA, revealing that the interfacial interaction of the alumina with the PLA matrix complicated the crystallization mechanism and growth geometry. 

Because the Avrami model is used for isothermal crystallization processes, the rate constant (*k*) was corrected using Equation (4):

(4) $$\log k_{c}=\frac{\log k}{\psi }$$

where *k_c_* is the corrected crystallization rate constant. This equation considers the effect of cooling rate under nonisothermal conditions. Table 3. shows that the value of *k_c_* increased with increasing cooling rate. Thus, the crystallization occurred faster at higher cooling rates. This completely agrees with the DSC, crystallization behavior results analyzed at different cooling rates.

### 4.4. XRD Analysis

**Figure 6:**
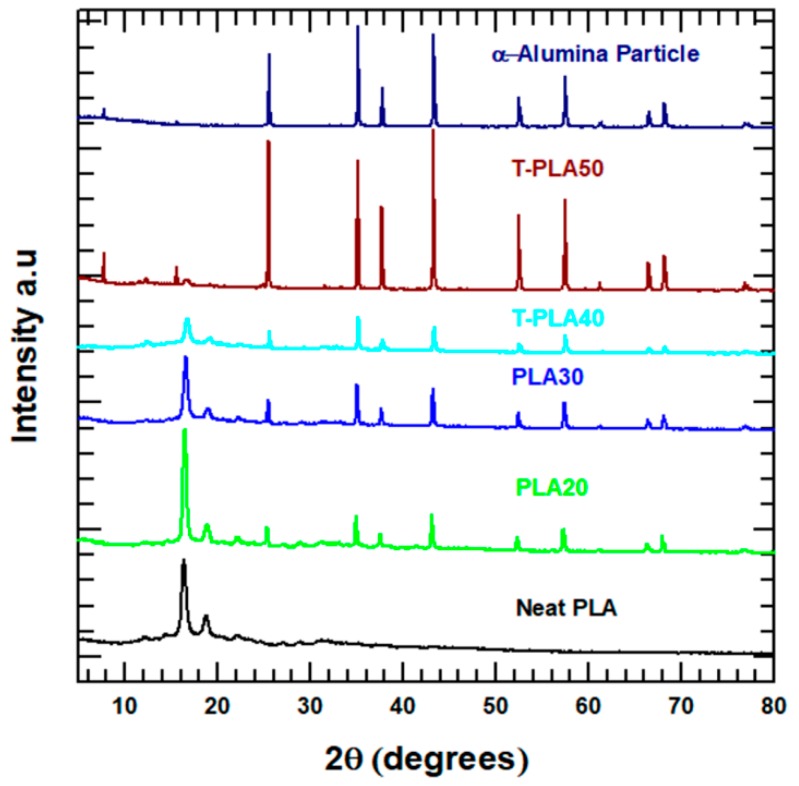
XRD patterns of the alumina and the PLA composites.

The crystalline nature and crystallization behaviors of the neat PLA and its hybrid composites were investigated by XRD analysis. The XRD patterns of the corresponding samples are presented in Figure 6. The XRD peaks of alumina reveal that the powder is highly crystalline and corresponds to single-phase α-Al_2_O_3_ [28]. The XRD pattern of the neat PLA shows high-intensity peaks at 16.4° and 18.87° and low-intensity peaks at 14.4° and 22.1°. The high-intensity peaks are associated with the reflections of the (110)/(200) and (203) planes, respectively, whereas the low-intensity peaks are assigned to the reflections of the (010) and (015) planes, respectively [6]. The highly crystalline plane at (110)/(200) mainly corresponds to the *α*-form crystals in the PLA matrix [29]. After the composite was fabricated with the incorporation of alumina in the PLA, the XRD peaks of the alumina increased in intensity with increasing filler loading, whereas the intensity of the diffraction peaks assigned to PLA became weaker. These results indicate that interaction between the PLA matrix and the hybrid fillers strongly affected the crystallinity of the hybrid PLA, consistent with the DSC results. Consequently, the incorporation of highly crystalline alumina increased the crystallinity of the PLA hybrid composites. 

### 4.5. Dynamic Mechanical Properties

**Figure 7:**
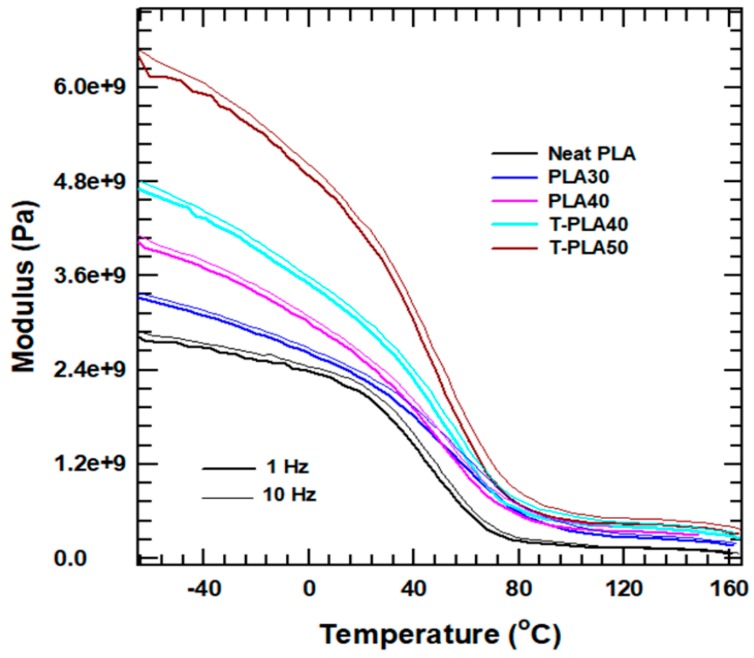
Storage modulus as a function of temperature at different frequencies.

The storage modulus of the PLA composites as a function of temperature was investigated at 1 and 10 Hz. The corresponding modulus curves are shown in Figure 7. The modulus showed a drastic increase at the starting temperature for the composites with higher filler loadings. At both 1 and 10 Hz, T-PLA50 had a storage modulus 2.25 times (125%) higher than that of the neat PLA. However, the plot clearly shows that the change in frequency did not strongly affect the modulus of the composites throughout the whole investigated temperature range. The T-PLA40 has a higher storage modulus than the PLA40, indicating that the treated fillers incorporated into the PLA matrix increased its rigidity because of the ability to restrict the mobility of PLA chains. 

The increase in the storage modulus of the PLA composite system fabricated with treated alumina indicated an improvement in the interaction between the PLA matrix and the treated alumina. This improved interaction confirming the restriction of the molecular mobility was the main effect that hindered the cold-crystallization process during the heating cycle [13]. The improvement of the storage modulus upon incorporation of treated crystal alumina into the PLA matrix is believed to be responsible for the increase in crystallinity. This result agrees with the results of the DSC experiments (Table 2). At temperatures greater than the glass-transition temperature, the storage moduli of all of the samples become almost equal irrespective of the frequency change. This observation is related to the chain relaxation of the PLA matrix at elevated temperatures. In general, DMA analysis confirmed that the incorporation of treated fillers hinders the mobility of the PLA matrix and makes the system more rigid, which is attributed to the decrease in intensity or disappearance of the cold-crystallization peak.

## 5. Conclusions

PLA is a widely utilized biodegradable and renewable polymer and a potential candidate to replace petrochemical polymers. In this work, PLA filled with treated and untreated alumina and a nano-AlN hybrid composite was synthesized via solution casting. The interaction of the hybrid fillers and the PLA matrix was analyzed using ATR-FTIR analysis. The crystallization and melting behaviors of the PLA hybrid composites were studied using DSC. The DSC results revealed that the hybrid composites exhibited a higher degree of crystallinity than the neat PLA. The complete crystallization of the treated PLA hybrid composites hindered cold crystallization during the second heating process. The crystallization kinetics were also studied using the Avrami model. The Avrami model parameters showed that the crystallization rate of PLA was affected by the inclusion of filler particles. The XRD results confirmed crystal formation upon the incorporation of fillers. The inclusion of nano-AlN and the increase in the crystallinity led to an improvement of the storage modulus. In general, this study confirmed that the filler loading, filler treatment and the change in heating and cooling rate have significant effect on the nonisothermal crystallization and degree of crystallinity of the PLA hybrid composites. 

## Figures and Tables

**Table 1:** Sample names and compositions of the composite samples.

Sample	Composition (wt %)		
	PLA	Alumina	AlN
Neat PLA	100	0	0
PLA20	80	18	2
T-PLA20	80	18 *	2
PLA30	70	28	2
PLA40	60	38	2
T-PLA40	60	38 *	2
T-PLA50	50	48 *	2

* The filler particle is Al–poly(MA).

**Table 2:** Melting and crystallization properties of the neat PLA and its hybrid composites.

*Sample*	*ψ (°C/min)*	*T_c_ (°C)*	*T_cc_ (°C)*	Δ*H_c_ (J/g)*	Δ*H_m_ (J/g)*	*X_c_ (%)*
*Neat PLA*	5	105.8	103.0	17.0	54.1	46.2
	10	105.7	108.2	7.6	51.8	33.4
	20	-	130.7	2.5	43.0	3.2
*PLA20*	5	109.0	-	29.9	41.2	55.3
	10	107.7	106.5	8.5	41.4	38.9
	20	106.1	114.0	11.8	37.0	21.8
*T-PLA20*	5	112.2	-	34.0	42.7	57.3
	10	106.0	96.1	20.8	46.8	58.8
	20	103.4	102.6	19.7	43.9	49.7
*PLA30*	5	110.0	-	29.7	37.2	57.1
	10	104.8	104.7	9.3	40.8	47.2
	20	103.4	114.1	5.2	31.3	9.8
*PLA40*	5	108.6	95.7	24.2	29.3	52.5
	10	105.8	105.8	10.0	37.8	47.1
	20	104.5	114.4	7.9	32.1	20.1
*T-PLA40*	5	111.2	-	23.6	41.3	71.1
	10	104.7	92.5	18.1	34.0	58.9
	20	101.0	98.2	17.6	40.5	58.3
*T-PLA50*	5	112.3	-	29.3	34.2	73.5
	10	106.5	91.6	13.6	28.7	56.9
	20	102.5	102.3	21.5	36.5	67.3

**Table 3:** Avrami parameters for each of the cooling rates.

*Sample*		*N*			*k (10^−3^)*			*k_c_ (10^−3^)*		
	*Ψ*	5	10	20	5	10	20	5	10	20
*Neat PLA*		2.56	2.72	2.49	10	262	159.1	401	875	102.3
*PLA20*		2.49	2.71	2.56	15	66	841	433	763	991
*T-PLA20*		2.72	3.92	2.85	15	25	142.5	432	691	101.8
*PLA30*		2.54	2.63	3.4	10	171	369	394	838	951
*PLA40*		2.90	2.64	2.63	5	113	107.8	342	804	100.4
*T-PLA40*		3.04	2.77	2.60	5	114	142.2	341	805	101.8
*T-PLA50*		2.73	2.63	3.04	13	304	985	422	888	999

## Supplementary Materials

The following are available online at https://www.mdpi.com/2073-4360/11/6/1077/s1.
