# Supplementary material for: Nonisothermal Crystallization of Surface-Treated Alumina and Aluminum Nitride-Filled Polylactic Acid Hybrid Composites
Source: Polymers (Basel). 2019 Jun 21;11(6):1077. doi: 10.3390/polym11061077 (PMC6630763; doi:10.3390/polym11061077)
Supplement: Supplementary file 1 [file polymers-11-01077-s001.pdf]

Supplementary material for

Nonisothermal crystallization of surface treated alumina and aluminum nitride filled polylactic acid hybrid composites

Zelalem Lule and Jooheon Kim*

School of Chemical Engineering & Materials Science,

Chung-Ang University, Seoul 156-756, Republic of Korea

*Corresponding author: Jooheon Kim, Tel:+82-2-820-5763; Fax: +82-2-812-3495; E-mail address: jooheonkim@cau.ac.kr (J. Kim)

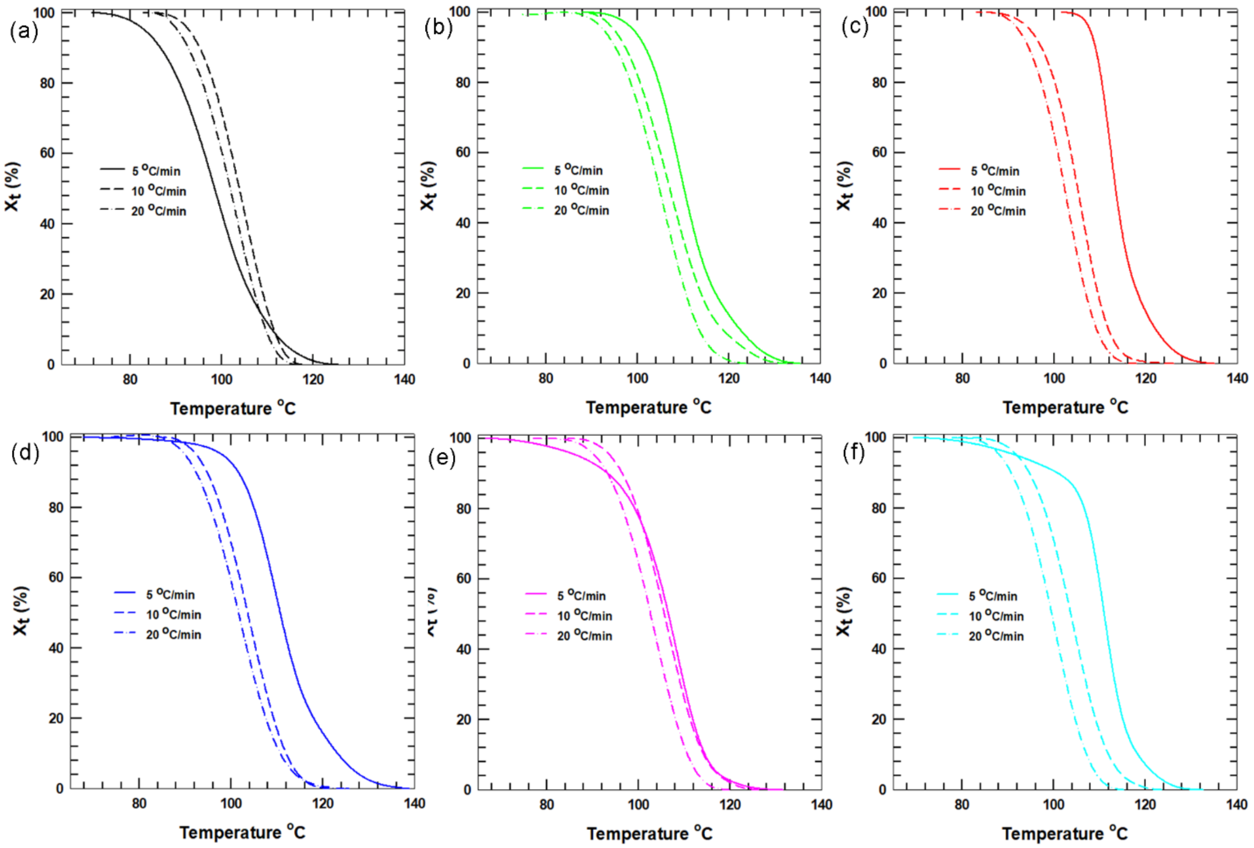

Figure S1. Relative crystallinity curve as a function of temperature: (a) neat PLA, (b) PLA20, (c) T-PLA20, (d) PLA30, (e) PLA40 and (f) T-PLA40.

At a cooling rate of 5°C/min, the crystallization started (onset) at a higher temperature and completed (offset) at a lower temperature compared with the crystallization processes at the other cooling rates. These results clarify the appearance of a broader crystallization curve (Fig. 3a–c) at lower cooling rates. However, the crystal formation temperature range in T-PLA50 was similar irrespective of the cooling rate, confirming that the interaction of the treated hybrid fillers and the PLA matrix influenced the crystallization rate and mechanism.

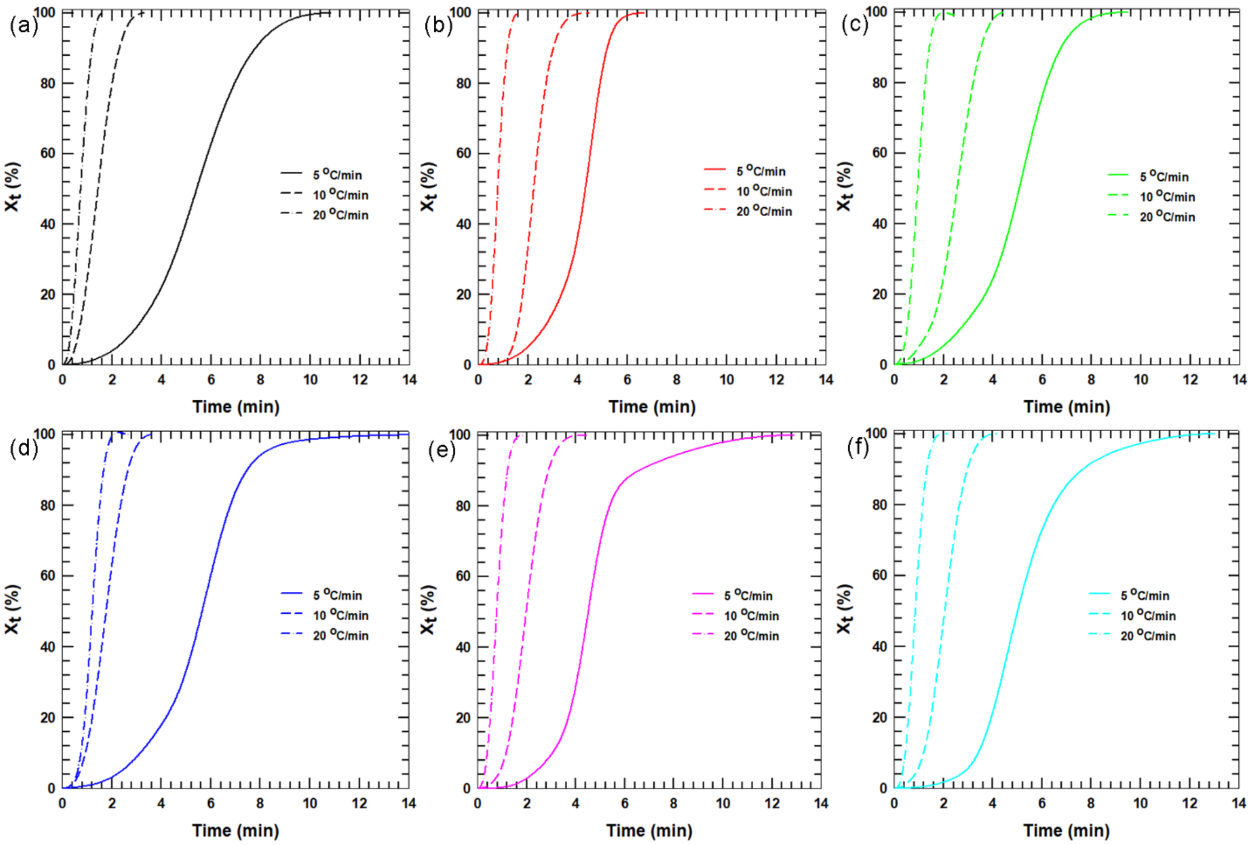

Figure S2. Relative crystallinity curve as a function of temperature: (a) neat PLA, (b) PLA20, (c) T-PLA20, (d) PLA30, (e) PLA40 and (f) T-PLA40.

Samples that crystallize at a lower cooling rate have sufficient time to undergo complete crystallization. Hence, either no cold-crystallization peak or a small cold-crystallization peak would be observed when the sample is heated. By contrast, if the sample is cooled at a high cooling rate, it would either uncrystallize or undergo partial crystallization, leading to the appearance of a cold-crystallization peak when the sample is heated.

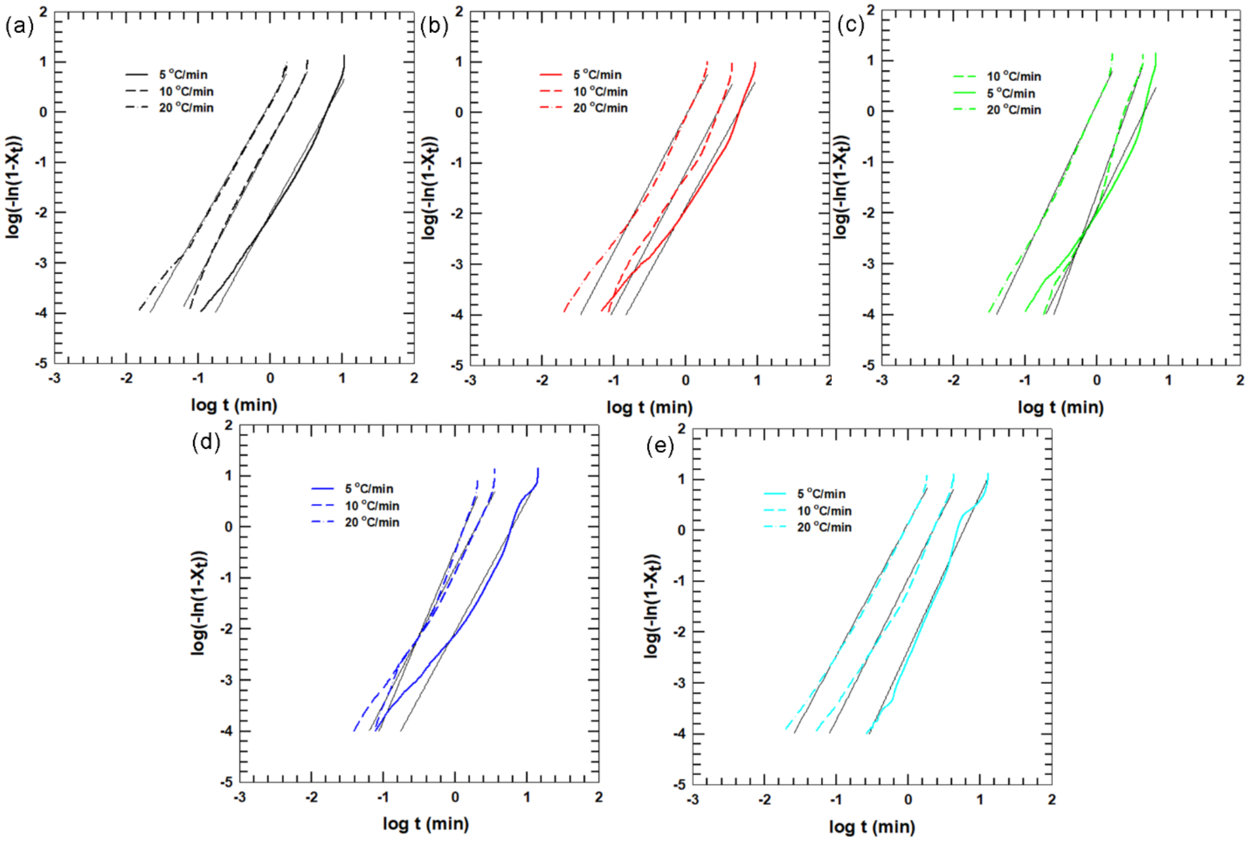

Figure S3. Avrami curves for (a) neat PLA; (b) PLA20; (c) T-PLA20, (d) PLA30 and (e) T-PLA40.
